# TSH Measurement and Its Implications for Personalised Clinical Decision-Making

**DOI:** 10.1155/2012/438037

**Published:** 2012-12-09

**Authors:** Rudolf Hoermann, John E. M. Midgley

**Affiliations:** ^1^Department of Nuclear Medicine, Klinikum Luedenscheid, Paulmannshoeherstraße 14, 58515 Luedenscheid, Germany; ^2^Consultancy Division, North Lakes Clinical, 6 High Wheatley, Ilkley, West Yorkshire LS29 8RX, UK

## Abstract

Advances in assay technology have promoted thyrotropin (TSH) measurements from participation in a multi-analyte assessment of thyroid function to a statistically defined screening parameter in its own right. While this approach has been successful in many ways, it has some grave limitations. This includes the basic question of what constitutes an agreed reference range and the fact that the population-based reference range by far exceeds the variation of the intraindividual set point. Both problems result in a potential misdiagnosis of normal and pathological thyroid function in a substantial proportion of patients. From a physiological perspective, TSH plays an integrated role in thyroid homeostasis. Few attempts have been made to adopt physiological insights into thyroid homeostasis for medical decision-making. Some emerging novel findings question the widely assumed log-linear TSH-FT_4_ relationship over the entire thyroid function spectrum. This data favours more complex hierarchically structured models. With a better understanding of its role in thyroid homeostasis in thyroid health and disease, TSH can be revisited in the context of thyroid regulation. This, in turn, could help overcome some of the limitations arising from its isolated statistical use and offer new prospects towards a more personalised interpretation of thyroid test results.

## 1. Background

Recognition of the existence of the entity later described as thyroid-stimulating hormone (TSH) was first evident in the late 19th century [1]. In the late 1960s and early 70s, academic immunoassays were developed and though initially cumbersome in use and somewhat insensitive, were quickly adopted as a diagnostic tool chiefly for determination of the hypothyroid state. From the mid 1980s to 1990s, steady developments in both convenience and sensitivity have so refined TSH assays that the concentrations of the hormone in subjects with normal thyroid function or with thyroid diseases are now readily quantifiable [2]. With the introduction of the third generation of commercial TSH assays into clinical routine diagnosis, assay sensitivity increased to 0.01 mU/L, allowing a clear-cut discrimination of the normal thyroid state from both hypothyroidism and hyperthyroidism [3].

In the present paper, we address the current use of TSH as the dominant parameter in thyroid function testing, explain some major limitations of this approach, and attempt to suggest areas of possible improvement. The fact that TSH is a measure of regulatory control with widely varying set-points among individuals rather than an outcome variable with a tight uniform range has various consequences for the statistical interpretation of this parameter, which differs sharply from other laboratory values. This, in turn, has further diagnostic, therapeutic, and prognostic implications that will be discussed. Improvements seem possible by respecting the regulatory individuality that is inherent in the parameter, as opposed to a population-based statistical use, and by advancing an understanding of the regulatory process and the interrelationship of TSH with circulating free thyroid hormones.

## 2. Current Diagnostic Strategy

Based on the methodological advances in TSH determination, the parameter has progressively evolved from its early adoption as an adjunct to the measurement of thyroid hormones to an exclusive parameter in its own right and consequently now dominates thyroid function testing. Modern diagnostic strategies have accordingly become heavily reliant on TSH measurement [2]. They have attributed various roles to TSH measurement as a screening tool, a therapeutic target in thyroid hormone treatment, and a prognostic marker [5–12]. With current disease classification based on TSH, this has introduced the subclinical states of hyperthyroidism or hypothyroidism that are defined by an abnormal TSH value in the presence of FT_4_ and FT_3_ values that still lay within their respective reference limits.

While this approach has been successful in many ways, it has also shifted the focus of TSH from its reactive and interactive role with thyroid hormones to an exclusive statistical parameter whose value is assumed to define the functional state of the subject. The present paper attempts to address some of the consequences of this paradigm shift and to assess some future perspectives for clinical decision-making.

## 3. Use and Limitations of TSH as a Statistical Parameter

While most problems of TSH measurement have been successfully resolved from the point of view of assay development and the analytical goals have been well defined, important issues that relate to the clinical application of the method still remain unsettled [13]. The assay performance does not resolve the problem that the immunological activity of TSH determined by the methods may not equate fully with its bioactivity [14]. This is important to note, because the bioactivity of TSH is subject to some variation depending on the level of thyroid function. This is a result of changes in carbohydrate content of the TSH molecule that is effected by the shift in function from hypothyroidism to hyperthyroidism [15]. Clinically, however, a slight dissociation between immunological and biological activity appears of minor significance, and there are currently no alternatives available to the immunoassays for routine clinical purpose. 

**Figure 1:**
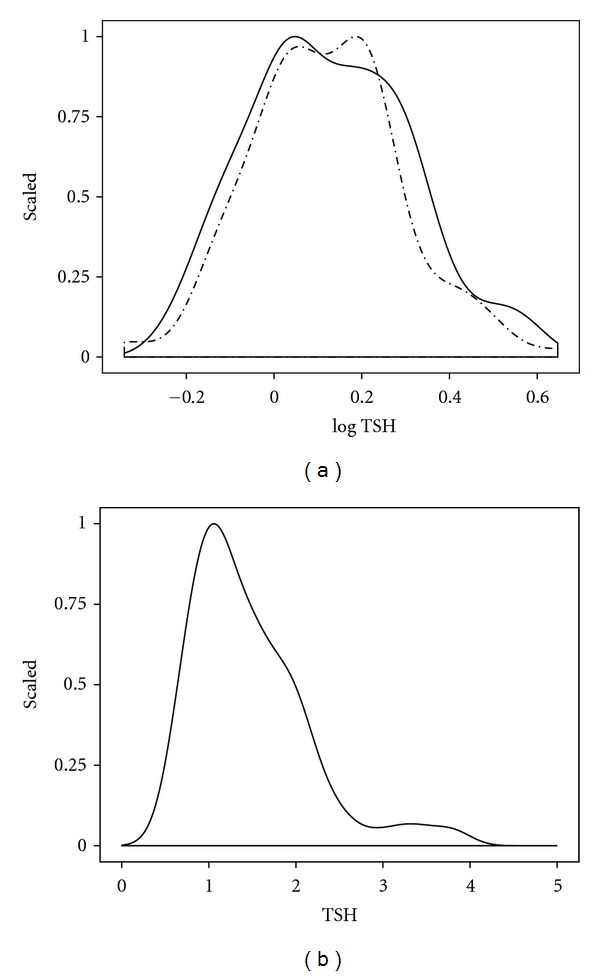
Distribution of log TSH (a) and TSH (b) in a clinical sample, compared to a random distribution. The distribution (solid line) was obtained from TSH measurements in 150 euthyroid subjects; a random distribution ((a), broken line) artificially generated by taking the mean and standard deviation of the sample.

This is less of a problem than the very basic question of what constitutes an agreed reference range. There has been a broad debate on the issue, particularly the setting of the upper reference limit, in which some authors argue for a wider reference range of approximately 0.3 to 4 mU/L and others advocate a more narrow interval with an upper limit of 2 mU/L [16–18]. The current state of affairs has recently been reviewed by Laurberg et al. [19]. It has also been pointed out that normal ranges and reference ranges are not necessarily to be considered as equivalent [20]. Importantly, TSH values are not normally distributed in a population, rather displaying a pattern of logarithmic normalisation and a skewed distribution. The latter findings have been questioned by arguing that if subjects with subtle thyroid disorders are included in the reference population, this may have distorted the observed normal distribution [21]. However, even when disease-free reference collectives were used, the disagreements still remain [22, 23]. Solely for illustration of the problem, but not for the support of assay validation per se, we have depicted the distribution of log TSH values that were obtained in a sample of euthyroid subjects (Figure 1(a)). The log transformation is generally required, because TSH values are not normally distributed (Figure 1(b)). For comparison, the normal distribution that was randomly generated from the mean and standard deviation of the sample is also shown (Figure 1(a)). The issue arises from the apparent distortion observed at the upper region of the TSH spectrum that has been frequently described, but not convincingly explained [21–23]. If one were to attribute the discrepancy to diseases such as unrecognized thyroid autoimmunity, a case could be made to adjust the spread of the normal distribution and consequently lower the upper reference limit. This is, however, not agreed nor is the reference interval [16–25]. 

The seemingly simple question of the disputed reference interval has far-reaching ramifications, which have been shown for instance in a study by Völzke et al. [24]. Employing a reference range established in their own investigation, according to the manufacturer's recommendation or as suggested by a US study [25], the authors reported a dramatic change in disease prevalence, from 7.7% to 24% in the case of diagnosed hypothyroidism and 0.8% to 3% for hyperthyroidism. This illustrates the grave consequences of the disagreement about TSH reference intervals that may result in clinical misclassification of a large proportion of patients.

## 4. Possible Diagnostic Solutions

**Figure 2:**
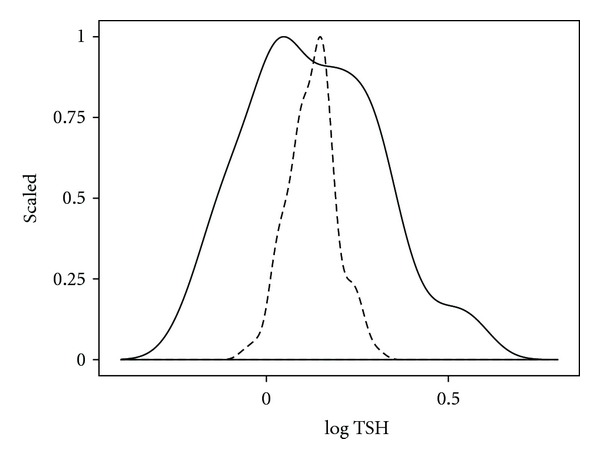
Distribution of log TSH in a group versus an individual. The TSH distribution in the group (solid line) was derived from 150 euthyroid subjects, with the individual spread (broken line) assumed to comprise 50% of the group variation (according to Andersen et al. [4]).

A way out of the dilemma posited above could be envisaged by prospectively correlating TSH measurements to clinical outcome measures. There have indeed been a number of studies that relate a TSH screening value to various future events such as cardiovascular disease, particularly atrial fibrillation, osteoporosis, psychiatric disorders, and mortality [27–30]. However, while the statistical diagnostic approach may be appropriate on a large scale in a population study, it appears unsuitable for guiding clinical decisions in an individual patient. Andersen and colleagues found the thyroid hormone concentrations in normal subjects to vary much less in an individual than in the population [4]. Given the magnitude of this variation of up to 50%, the distinction between a normal and pathological value cannot rely on the population-defined reference, but has to account for the individual patient's normal set point within the individual laboratory's reference range [4]. The high ratio of interindividual to intraindividual variability conflicts with any efforts to define a universally applicable clear cut-off and considerably limits the usefulness of the population-based reference values for clinical decision-making. This problem can be graphically illustrated (Figure 2). The figure shows the distribution of log TSH in 150 euthyroid subjects that resembles the reference range in a population and, for comparison, the distribution that results when reducing the standard deviation by 50%, which equals the spread of a normal intraindividual distribution according to Andersen et al. [4]. The movement of the narrow bell-shaped curve within the broader bell curve reflects the uncertainty about “true” normality that is inherent in the application of a population-based TSH reference system. This uncertainty on an individual basis exceeds by far that of many other laboratory parameters. Consequently, this makes the need for an individualised approach towards thyroid function testing more compelling than in other conditions of the subjects.

Another limiting factor is the significant fluctuation of the TSH levels over the course of time, which has been reported to occur in subclinical hypothyroidism [31]. The TSH value has, therefore, to change by at least 30% to discriminate between a natural variation and a real progression [31]. Additional influences such as gender, age, or time of sampling are less pronounced [32]. Also, in predictive long-term studies that have relied on a single initial TSH value this may not be an integer of the thyroid function status over the whole time period, but subject to change, as the dysfunction could have worsened or improved since the measurement was obtained [33]. Interestingly, monitoring intervals have been shown to directly influence the outcome [34]. Conditions that directly alter or interfere with the reliability of thyroid test results such as severe nonthyroid illness, pregnancy, severe renal insufficiency, and pituitary disorders are another topic [35]. 

The issue of thyroid hormone measurement in pregnancy has only recently been addressed by two updated guidelines issued by the American Thyroid Association in conjunction with the American Society of Clinical Endocrinologists and independently by the Endocrine Society following one year earlier pregnancy-specific guidelines [5, 36, 37]. Pregnancy exemplifies a choice between FT_4_ measurement and reliance on measuring total T_4_ (TT_4_), corrected for transport proteins, predominantly TBG. The new guidelines tend to favour TT_4_ measurement over FT_4_ methods, with one of them making this an explicit recommendation [5]. FT_4_ immunoassay measurement has historically been perceived as inaccurate in pregnant women by many authors [5, 36, 37]. However, when scrutinized more closely, the assumption of a superiority of a TT_4_-TBG combination in pregnancy compared to FT_4_, despite acknowledged limitations in assay validation, does not hold true. In pregnancy, a combination of TBG and TT_4_ confers a variation to the reference range that is by far greater, compared to direct FT_4_ measurement, and therefore less accurate in defining the “normal” range in this situation in a patient. Spreads can be compared by the standardized variation (SD/Mean) or the ratio of the 97.5 percentile to the 2.5 percentile of the range [38]. In one study, when calculating the values on the basis of the generally accepted ranges for the parameters, the ratio was 3.0 in TT_4_ and only 2.4 in FT_4_ [38, 39]. The smaller spread for FT_4_ suggests that the TT_4_ reference range is compromised by uncorrected TBG variation [38]. Since TBG values are higher in pregnancy, the effect of their variation has a greater impact in this condition, but it is equally true for the nonpregnant situation. Hence, the choice here is between measurement of TT_4_, which is clearly diagnostically inferior, or FT_4_, which sometimes lacks careful validation among different manufacturers as a prerequisite to its adequate use. The general reliability of FT_4_ results obtained with the assays during pregnancy has, however, been verified by comparison with equilibrium dialysis ID-LC/tandem MS candidate reference measurement [40].

We would prefer an FT_4_ method applied with methodological rigor and careful attention to range setting and consider the novel recommendation to measure TT_4_ in pregnancy an ill-advised backward step, apart from practical considerations that most routine laboratory by now have wide experience with FT_4_ assays, but not TT_4_. FT_4_ should be used in conjunction with TSH, and interpretation in pregnancy must be based on gestation time-specific reference intervals of the two parameters and with recognition of variability in assay performance [5, 37, 38, 41]. A lack of availability and clinical experience currently limit the use of novel mass-spectrometric (LC-MS/MS) techniques [37, 42]. Though inconsistency of assay performance is a recognised obstacle to optimal diagnostic protocols, a full discussion of assay validation is beyond the scope of this paper [43, 44]. 

Although the limitations of the current use of TSH measurement have been well documented and subject to a number of excellent reviews, we generally lack suggestions as to how to improve clinical management strategies. Obviously, a desirable aim would be to move from a population-based statistical perspective to a more personalised approach. While, to an extent, this seems to be what we aim at in good clinical practise when considering the whole picture including laboratory tests, clinical examination, and subjective well-being of the patient, it does not appear to be adequately reflected in current evidence-based guidelines [5].

We conclude that advances in assay techniques have unduly promoted TSH measurement to its current role as an exclusive statistical estimate in its own right and the most important single parameter in thyroid function testing, thereby optimising both convenience and cost. However, the predominant use of TSH as a statistical parameter has some severe shortcomings that limit its clinical usefulness in a given patient. A revision may be needed to reconcile TSH measurement with the challenge of not only evidence-based but personalised medicine.

## 5. Role of TSH as a Physiological Parameter

From a physiological perspective, TSH is not an isolated player, but an integrated part in a complex system of thyroid homeostasis. Thyroid homeostasis is maintained by the negative feedback that is given by the peripheral concentrations of the hormones FT_4_ and FT_3_ to regulatory centres in the hypothalamus and pituitary gland that govern thyroid hormone production via the adjusted release of a thyroid-stimulating hormone (TSH) into the circulation. A better understanding of thyroid hormone homeostasis including the role of TSH in the context may therefore aid in improving the diagnostic reliability of TSH measurement from both a methodological and clinical perspective. Modern thyroid function testing has the danger of exploiting TSH feedback out of context and has replaced the endpoint-based definition (measuring FT_4_ and FT_3_) by a TSH-centred protocol, as discussed above. This paradigm shift has made interpretation of test results seemingly easy. From a homeostatic perspective, however, dysfunctional states, such as hypothyroidism or hyperthyroidism present adaptive challenges to the homeostatic system that result in a complex and concerted pattern of actions to restore euthyroidism or at least ameliorate the situation. This approach sees TSH as an integral part of the homeostatic system rather than a separate or even dominant determinant.

**Figure 3:**
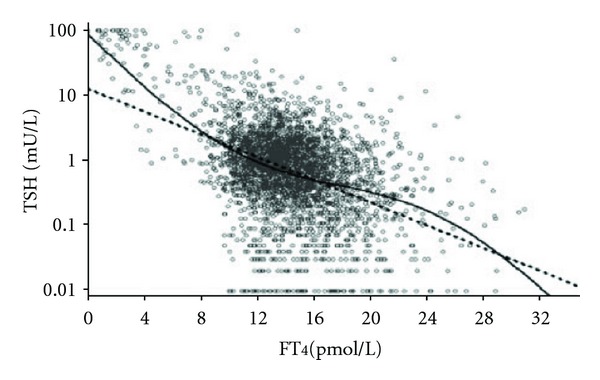
Comparison of a nonlinear model with the log linear standard model in a large clinical data set. The superior fit of the non-linear model was proven by comparative curve fitting, as shown in [26]. The figure is reprinted by permission of the publisher Bioscientia (http://www.eje-online.org/site/misc/permissions_commercial_reprints.xhtml).

Attempts have been made to adopt physiological insights into thyroid homeostasis for medical decision-making. Some authors have developed fundamental mathematical models of thyroid homeostasis; others have exploited the interplay of FT_3_, FT_4_, and TSH to reduce the wide spread of exclusive TSH measurements [45–48]. Earlier studies have shown that FT_4_ has a relatively more pronounced influence on pituitary TSH secretion than does FT_3_ [49]. This is somewhat paradoxical, because FT_3_ is the more biologically active hormone of the two, and T_4_ only gains its activity following its deiodination, a process known as conversion and regulated by various deiodinases [49]. The phenomenon has been explained by the specific properties of pituitary deiodinase type 2 which allows efficient conversion to continue with high FT_4_ concentrations at the central levels, whereas a downregulation of the enzyme occurs in peripheral tissues [49]. The natural relationship between TSH and FT_4_ has been widely assumed to be both universal over the whole thyroid function spectrum and log linear [50–53]. A recent study has challenged this widely held view of a single all-encompassing log linear correlation between TSH and FT_4_ [26]. In this study, we demonstrated a more complex nonlinear interrelationship between the two parameters in a large clinical sample (Figure 3) [26]. While there has been some criticism as to the retrospective study design, heterogeneous study population, and reliability of FT_4_ measurement, the findings have recently been independently confirmed [54, 55]. If the relationship is truly non-linear and noncontinuous, the system of thyroid hormone feedback control has to be reconsidered, because it is critical to the current role of TSH, for instance, when used as an exclusive therapeutic target establishing dose adequacy in patients treated with exogeneous L-T_4_.

## 6. Implications for Regulation and Diagnostic Strategies

Taken together, the studies described have several implications for our understanding of both hypothalamic pituitary thyroid regulation and the clinical interpretation of TSH results. Firstly, they suggest that a simple log linear correlation may not reliably represent the relationship between TSH and FT_4_. Hence, understanding of the interplay of TSH and FT_4_ requires more complex modelling. Secondly, they suggest that pituitary thyroid feedback control is not convincingly represented by an all-encompassing single process, but better characterised by a hierarchically structured type of control with distinct patterns of operative mechanisms that are unique to different functional states. 

This assumption is further supported when reviewing the literature on the molecular aspects of thyroid hormone regulation. Earlier studies have shown the existence of isoforms of thyroid hormone receptors (TR) in different tissues [56]. TRß2 is exclusively expressed in the central nervous system including the pituitary and shows an up to 10-fold enhanced sensitivity to thyroid hormones [57–59]. Consequently, the pituitary is able to sensitively monitor small increases in T_4_ supply and to counteract T_4_ overproduction in advance of the occurrence of any effect on the peripheral tissues. Basically, TSH dampens its own action in anticipation of its effect on T_4_ secretion via an ultrashort feedback loop, thereby also giving rise to a pulsatile secretory pattern [26, 46, 60]. The activities and contributions of various deiodinases controlling conversion of T_3_ from T_4_ have also been reported to differ in the hypothyroid and hyperthyroid state [61]. TRH is key in orchestrating a response in the event of hormone undersupply. It stimulates pituitary TSH secretion and, additionally, modulates its bioactivity [15, 62]. Studies with transgenic animals have demonstrated regulatory varying roles for both thyroid hormone feedback and TRH [63, 64]. The distinct mechanisms unravelled by the molecular studies are difficult to reconcile with the assumption of a single all-encompassing gradient of the relationship between TSH and FT_4_, but would naturally explain a complex and hierarchical pattern of the regulation. It also implies that correlating a given TSH value and FT_4_ level may not be a simple and straightforward process, as previously thought.

## 7. Clinical Perspective of TSH Measurement towards a Personalised Approach

The promotion of TSH into a parameter of its own right, with a statistical distribution and correlation to clinical outcomes and targets, has on the one hand greatly facilitated thyroid function testing. On the other hand, it has failed to consider adequately the individual set points of the pituitary thyroid axis that are far more narrowly defined than the broad interindividual distribution of the parameter (Figure 2). This failure may have consequences for clinical decision-making, in terms of not only misclassification of health and disease, as mentioned above, but also misjudging dose adequacy in thyroid hormone replacement. The latter is in particular need of being addressed because clinical studies in treated patients have invariably demonstrated a disturbingly high rate of patients that were dissatisfied with their mode of treatment [65, 66]. Questions such as of T_4_ monotherapy versus T_3_/T_4_ combination treatment, we believe, cannot be assessed on statistical grounds alone, without a solid understanding of the underlying regulatory process. The therapeutic requirements may differ in different conditions or populations, and a slightly elevated TSH that is indicative of subclinical hypothyroidism per definition cannot be used as a universal marker, because it has been reported to increase mortality in some populations, whereas it promoted longevity in others, particularly in the elderly [11, 12, 67, 68]. 

A remedy may be to revisit the roots of TSH determination that has originated as a thyroid feedback regulator in thyroid homeostasis. While studies devoted exclusively to TSH abound in the literature, the interrelationship of thyroid parameters and their homeostatic roles have received scant attention [26, 46–55, 69–71]. Even when both TSH and thyroid hormone measurements were available on a large scale, as was for instance the case in a recent study on cardiovascular mortality, their use was limited to the purpose of defining subclinical states of thyroid dysfunction, but did not include a broader and more detailed analysis of the interplay of the parameters on the outcome [11, 72]. The latter approach seems more liable to advance our understanding and propel TSH measurement towards a more personalised approach of thyroid function testing. It does, however, require as a prerequisite a better understanding of thyroid hormone feedback regulation, which is currently lacking, but is evolving in new directions [26, 54, 55]. The new models allow for distinctly different adaptive homeostatic processes to be activated in either euthyroidism, hypothyroidism, or hyperthyroidism. Each state is thereby defined as a qualitatively different entity with a distinct pattern of homeostatically operative mechanisms and equilibria. 

A dynamic TSH response that depends on the prevailing thyroid function state closely links the parameter to the circulating thyroid hormones, mainly FT_4_. Genetic variability may also play a role in shaping the relationship, for example, via deiodinase polymorphisms [70, 71]. 

**Figure 4:**
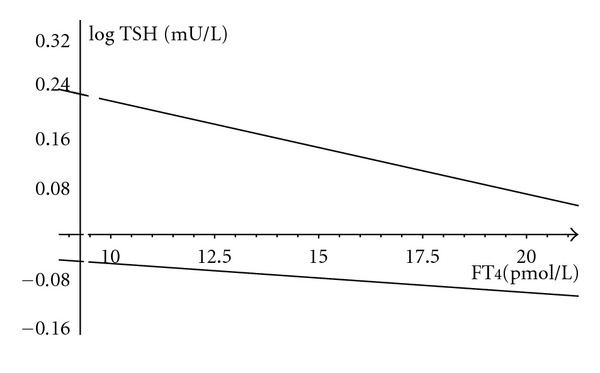
FT_4_ adjusted TSH band in the euthyroid range. The linear regression fitted to the euthyroid sample (*n* = 150) is described by the equation (±SE), log TSH = −0.014 (±0.006) ∗ FT_4_ + 0.36 (±0.1). The lines indicate the upper and lower 2 SD range of the regression line.

An FT_4_-corrected TSH could, therefore, reduce the variation of test results. The complex nature of the FT_4_-TSH relationship requires more advanced algorithms to be established and clinically tested. A simplified tentative representation of the idea is shown in Figure 4, based on a fitted log linear model to the euthyroid sample. Smaller pilot studies in clinical settings have indicated that a correlated calibration of TSH and FT_4_ or FT_3_ measurements outperforms the standard procedure in reducing the diagnostic spread [47, 48]. Jostel et al. [47] have demonstrated that an FT_4_-adjusted TSH, termed TSH index by the authors and calculated according to the formula TSHI = log TSH + 0.13 ∗ FT_4_, facilitated the diagnosis of secondary hypothyroidism, providing an accurate estimate of the severity of TSH deficiency in hypopituitarism. The TSH index has not been extensively validated in primary thyroid disorders [47]. Meier et al. [48] introduced a bivariate zonal representation for the normal ranges of thyroid function tests. The authors validated the method in 257 volunteers and demonstrated an improved diagnostic accuracy, based on TSH-FT_3_ zones, in borderline cases, compared to the uncorrelated standard approach using the normal distributions of TSH and FT_3_ [48]. Complex cybernetic models could also tie FT_4_ and TSH together in a more accurate manner, to establish regulation-based discriminatory cut-offs of the parameters and increase their diagnostic value, but these have not been broadly tested either [45, 46]. 

In summary, correlative studies are few, compared to the vast literature on TSH. As a result, they have failed to gain broader acceptance and did not include more elaborate modelling. 

The availability of presumably more reliable assays for FT_4_ based on LC-MS/MS may be advantageous for the development of such ideas [42, 53, 73]. However, evaluating FT_4_ assays and comparing different methods by conflating sera encompassing the whole spectrum of thyroid function, as has been proposed by some authors [53, 73], is not supported in our view, given that the three physiological areas have their own responses and any necessarily arbitrary mixture of subjects must have a distorting influence on the results. Despite some promising reports, there are no large trials currently available to reliably assess the clinical improvement introduced by the new methods, compared to existing technology. 

In this setting, TSH screening could still have an important role as a first line test, in order to sensitively detect abnormalities, but the therapeutic decision should be made more specifically by interpreting values in the context of the underlying conditions and homeostatic equilibria. A slightly elevated TSH in subclinical hypothyroidism may accompany the successful adaptive response in some patients, but signal a failed adaption in others. Prognostic studies do not support an indiscriminate use of TSH as a therapeutic target [12, 67]. Elevated TSH in subclinical hypothyroidism has been implicated to signal an adverse prognostic outcome in large population studies, but has also been documented as a marker of longevity in a very aged population [11, 12, 67]. This discrepancy argues against the adoption of a simplistic universal use of TSH values that are slightly outside the reference range, but are also taken out of context to the individual or particular situation. While interesting statistical associations have been documented between TSH and various clinical outcomes, a deeper understanding at the physiological and regulatory level is required to put them into context and improve clinical decision-making.

## 8. Summary

The current mainly statistically based use of TSH in thyroid function testing has some severe limitations, including the problem of nonexisting agreed reference limits, a lack of consideration of individual set points, and a prognostic heterogeneity in different populations. In the light of recent molecular and clinical evidence, revised and more refined modelling of the interrelations of TSH and thyroid hormones appears to be both a physiological requirement and a promising avenue towards an overdue reevaluation of TSH as an exclusive diagnostic standard and therapeutic target. Based on studies on the FT_4_-TSH relationship including our own work and a review of the molecular mechanisms described in the literature we propose a complex non-linear model and hierarchical structure of the thyroid hormone TSH interaction. We promote the interpretation of TSH results in close correlation to the thyroid hormone milieu to reduce the uncertainty of interpretation. While a few smaller studies could demonstrate a conceptually higher accuracy, compared to the standard diagnostic procedure, a possible broader application of such concepts awaits further methodological and clinical evaluation.
